# Spatiotemporal GLP-1 and GIP receptor signaling and trafficking/recycling dynamics induced by selected receptor mono- and dual-agonists

**DOI:** 10.1016/j.molmet.2021.101181

**Published:** 2021-02-06

**Authors:** Aaron Novikoff, Shannon L. O'Brien, Miriam Bernecker, Gerald Grandl, Maximilian Kleinert, Patrick J. Knerr, Kerstin Stemmer, Martin Klingenspor, Anja Zeigerer, Richard DiMarchi, Matthias H. Tschöp, Brian Finan, Davide Calebiro, Timo D. Müller

**Affiliations:** 1 Institute for Diabetes and Obesity, Helmholtz Diabetes Center, Helmholtz Zentrum München, Neuherberg, Germany; 2 German Center for Diabetes Research (DZD), Neuherberg, Germany; 3 Division of Metabolic Diseases, Department of Medicine, Technische Universität München, D-80333 Munich, Germany; 4 Institute of Metabolism and Systems Research, University of Birmingham, Birmingham, B15 2TT, UK; 5 Center of Membrane Proteins and Receptors (COMPARE), Universities of Nottingham and Birmingham, Birmingham, B15 2TT, UK; 6 Novo Nordisk Research Center Indianapolis, Indianapolis, IN 46241, USA; 7 Chair for Molecular Nutritional Medicine, School of Life Sciences, Technical University of Munich, 85354 Freising, Germany; 8 Institute for Diabetes and Cancer, Helmholtz Center Munich, 85764 Neuherberg, Germany; 9 Joint Heidelberg-IDC Translational Diabetes Program, Inner Medicine 1, Heidelberg University Hospital, Heidelberg, Germany; 10 Department of Chemistry, Indiana University, Bloomington, IN 47405, USA; 11 Helmholtz Zentrum München, Neuherberg, Germany; 12 Technische Universität München, München, Germany; 13 Department of Pharmacology and Experimental Therapy, Institute of Experimental and Clinical Pharmacology and Toxicology, Eberhard Karls University Hospitals and Clinics, 72076 Tübingen, Germany

**Keywords:** GLP-1R, GIPR, Biased agonism, Receptor Internalization, Receptor Trafficking, Dual-agonists

## Abstract

**Objective:**

We assessed the spatiotemporal GLP-1 and GIP receptor signaling, trafficking, and recycling dynamics of GIPR mono-agonists, GLP-1R mono-agonists including semaglutide, and GLP-1/GIP dual-agonists MAR709 and tirzepatide.

**Methods:**

Receptor G protein recruitment and internalization/trafficking dynamics were assessed using bioluminescence resonance energy transfer (BRET)-based technology and live-cell HILO microscopy.

**Results:**

Relative to native and acylated GLP-1 agonists, MAR709 and tirzepatide showed preserved maximal cAMP production despite partial Gα_s_ recruitment paralleled by diminished ligand-induced receptor internalization at both target receptors. Despite MAR709's lower internalization rate, GLP-1R co-localization with Rab11-associated recycling endosomes was not different between MAR709 and GLP-1R specific mono-agonists.

**Conclusions:**

Our data indicated that MAR709 and tirzepatide induce unique spatiotemporal GLP-1 and GIP receptor signaling, trafficking, and recycling dynamics relative to native peptides, semaglutide, and matched mono-agonist controls. These findings support the hypothesis that the structure of GLP-1/GIP dual-agonists confer a biased agonism that, in addition to its influence on intracellular signaling, uniquely modulates receptor trafficking.

## Highlights

• GLP-1/GIP dual-agonists, MAR709 and tirzepatide, are partial effectors at multiple GLP-1R pathways, yet retain full cAMP agonism.
• MAR709 elicits comparable GLP-1R incorporation into Rab11^+^ recycling endosomes relative to the native peptides and acyl-GLP-1.
• At the GIPR, both dual-agonists exhibit full-agonism properties with limited receptor internalization/trafficking properties.

## 1 Introduction

Glucagon-like peptide-1 (GLP-1) is a pleiotropic hormone with broad pharmacological potential due to its ability to improve body weight, food intake, and glucose metabolism [1]. However, active GLP-1, which is primarily GLP-1 (7–36 amide) and to a lower extent GLP-1 (7–37), is subject to rapid proteolytic degradation and fast renal elimination [[2], [3], [4], [5]]. Long-acting analogs with biochemical modifications in the GLP-1 sequence have been designed to overcome these limitations and are in clinical use for treating type 2 diabetes [6,7]. Despite molecular enhancements in time action, dose-dependent adverse effects limit the maximal efficacy and overall therapeutic potential of GLP-1R mono-agonists [8]. Single chimeric molecules with dual agonism at the receptors for GLP-1 and glucose-dependent insulinotropic polypeptide (GIP) improve body weight and glucose handling with superior potency to GLP-1R mono-agonists in preclinical [9,10] and clinical studies [11]. While GLP-1/GIP dual-agonists have advanced to phase 3 clinical trials for treating obesity and diabetes, the contribution of GIPR agonism to these applications is questionable. Mice with GIP receptor (GIPR) depletion are protected from diet-induced obesity [12], and patients with type 2 diabetes show an impaired insulinotropic response to GIP infusion [13]. Antibodies antagonizing GIPR improve body weight and glucose metabolism in obese rodents and non-human primates [14]. Recent hypotheses to reconcile these discrepancies include GIPR agonists acting as functional GIPR antagonists, or alternatively that specific ligands engage unique receptor signaling, trafficking, and/or recycling dynamics, commonly referred to as biased-agonism [15]. Biased agonism at the GLP-1R has been linked to differential cellular desensitization capacities via differences in receptor internalization and/or β-arrestin recruitment, as has been shown for Phe1-substituted exendin-4 [16]. In addition to the GLP-1R agonists exendin-4 and oxyntomodulin, both of which demonstrate bias toward β-arrestin recruitment [17], α/β amino acid modifications to the GLP-1 backbone sequence can also result in differential GLP-1R signaling [18]. Likewise, the GLP-1/GIP dual-agonist tirzepatide (LY3298176; Eli Lilly, Indianapolis, IN, USA) was recently reported to favor phosphorylation of ERK1/2 relative to β-arrestin and Gα_s_ protein at both target receptors [19].

The aim of this study was to assess the spatiotemporal GLP-1 and GIP receptor signaling, trafficking, and recycling dynamics mediated by select GLP-1R and GIPR mono- and dual-agonists. MAR709 is characterized as a balanced GLP-1/GIP co-agonist and is acylated with a C16 fatty mono-acid, which allows for once-daily time action in humans [20,21]. Tirzepatide is characterized as an imbalanced GLP-1/GIP co-agonist that favors GIPR potency and is acylated with a C20 fatty di-acid at position 20, which allows for once-weekly time action in humans [22]. Our results show that both dual-agonists, MAR709 and tirzepatide, act as partial effectors at GLP-1R for G protein recruitment, receptor internalization, β-arrestin recruitment, Rab5^+^/Rab7^+^ receptor trafficking, and endosomal G-protein recruitment, while retaining full-agonist capacity for cAMP production. Interestingly, despite showing a reduced receptor internalization rate, MAR709 acts as a full effector for stimulating GLP-1R incorporation into Rab11^+^ recycling endosomes. At the GIP receptor, dual-agonists similarly act as full agonists for cAMP, but lack the G protein recruitment partial agonism profile and display limited receptor internalization and trafficking properties. Our data support the hypothesis that biased agonism with unique receptor signaling and trafficking properties might be a potential basis for enhanced metabolic benefits of these GLP-1/GIP dual-agonists.

## 2 Methods

### 2.1 Plasmids

Untagged human GLP-1R was purchased from Sino Biological Inc. (Cat #: HG13944-UT, Beijing, China) and untagged human GIPR and human GIPR-turbo GFP were purchased from OriGene Technologies Inc. (Cat #: SC110906 and RG210811, Rockville, MD, USA). Human GLP-1R-GFP was a gift from Professor David Hodson (University of Birmingham, Birmingham, UK). Human GLP-1R-Rluc8 (hGLP-1R-Rluc8) was a gift from Professor Patrick Sexton (Monash University, Melbourne, Australia). Human CMV-GIPR-Rluc8 (hGIPR-Rluc8) with VSLGSSG residues was constructed and purchased from VectorBuilders Inc. (Neu-Isenburg, Germany). cAMP sensor pcDNA3L-His-CAMYEL (ATCC MBA-277TM) was purchased from ATCC (Manassas, VA, USA) [23]. NES-Nluc-mini-G plasmids (Gα_s_, Gα_q_, Gα_i_, and Gα_12/13_) and subcellular/endosomal markers Rab GTPases (Rabs) Venus-Rab5a (early endosomes), Venus-Rab7a (late endosomes/lysosomes), Venus-Rab11a (recycling endosomes), and Venus-KRAS (plasma membrane) were gifts from Kevin Pfleger (Harry Perkins Institute of Medical Research, Nedlands, WA, Australia) as originally published by Professor Nevin Lambert (Augusta University, Augusta, GA, USA) [24]. β-arrestin 1/2-Rluc8 plasmids were a gift from Professor Terry Hébert (McGill University, Montreal, Canada).

### 2.2 Peptide synthesis

Semaglutide was provided by Novo Nordisk (Bagsværd, Denmark). All of the other peptides were prepared via standard automated Fmoc/tBu solid-phase peptide synthesis on Rink Amide ChemMatrix resin. An orthogonal protecting group strategy was used to incorporate the protraction moiety onto the appropriate lysine side chain. Following synthesis, crude compounds were cleaved from the resin with 95:2.5:2.5 trifluoroacetic acid/water/triisopropylsilane. The crude compounds were purified by reversed-phase high-performance liquid chromatography (RP-HPLC) on a Luna C8 (2) preparative column with a gradient of water/acetonitrile containing 0.1% trifluoroacetic acid, then lyophilized to produce the desired compounds as white powders. Compound identity was confirmed via RP-HPLC-mass spectrometry. hGLP-1 (7–36 amide) was purchased from Anaspec (Cat #: AS-22463, Fremont, CA, USA). hGIP (1–42) was purchased from Anaspec (Cat #: AS-61226-1, Fremont, CA, USA).

### 2.3 Cell culture

HEK293T cells lacking endogenous GLP-1R and GIPR were cultured in Dulbecco's Modified Eagle Medium (DMEM, Cat #: 11995073, Life Technologies, Carlsbad, CA, USA) with 10% heat-inactivated fetal bovine serum (FBS, Cat #: 10500064, Life Technologies, Carlsbad, CA, USA), 100 IU/mL of penicillin, and 100 μg/mL of streptomycin solution (Pen-Strep, Cat #: P4333, Sigma–Aldrich, St. Louis, MO, USA). Min6 cells were cultured in Dulbecco's Modified Eagle Medium with 15% heat-inactivated fetal bovine serum, 100 IU/mL of penicillin, 100 μg/mL of streptomycin solution, 20 mM of HEPES, and 50 μM of β-mercaptoethanol. All of the cells were maintained at 37 °C in 5% CO2.

### 2.4 Ligand-induced BRET assay

The cells were seeded (700,000 cells/well) in 6-well plates and incubated to ∼70% confluency in complete media supplemented with 10% FBS and 1% Pen-Strep. Twenty-four hours after seeding, overexpression of target proteins was performed under transient transfection conditions using Lipofectamine 2000 (Cat #: 11668019, Invitrogen, Carlsbad, CA, USA) according to the manufacturer's protocol without including additional carrier DNA. Twenty-four hours after transfection, the cells were washed with PBS, then detached and resuspended in FluoroBrite phenol red-free complete media (Cat #: A1896701, Life Technologies, Carlsbad, CA, USA) containing 5% FBS and 2 mM of l-glutamine (Cat #: 25030081, Life Technologies, Carlsbad, CA, USA). Then 100,000 cells/well were plated into poly-d-lysine-coated (Cat #: P6403, Sigma–Aldrich, St. Louis, MO, USA) 96-well white polystyrene LumiNunc microplates (Cat #: 10072151, Thermo Fisher Scientific, Waltham, MA, USA). After 24 h, the media was replaced with PBS (Cat #: 10010056, Gibco, Carlsbad, CA, USA) containing 10 μM of coelenterazine-h (Cat #: S2011, Promega, Madison, WI, USA) or 1:500 NanoGlo (Cat #: N1110, Promega, Madison, WI, USA). BRET^1^ measurements were taken every 30 s for 2 min at 37 °C using a PHERAstar FS multi-mode microplate reader with 430–485 nm and 505–590 nm dual filters. Baseline measurements were taken after 5 min of incubation with coelenterazine-h or NanoGlo. The cells were then treated with a vehicle or the respective agonists. The resulting ratiometric BRET signal between the interacting fluorophore and lumiphore was normalized by subtracting the background ratio (505–590 nm emission over 430–485 nm) of the vehicle-treated wells with the matched agonist-treated wells producing a signal defined as the “ligand-induced BRET ratio” [25]. The temporal data of the vehicle-corrected agonist measurement was then normalized to the baseline reading of the same well. The first BRET reading following treatment with agonist/vehicle was the subsequent measurement after the zero time point. Positive or negative incremental areas under the curves (+iAUC/-iAUC) were calculated where noted. Each experiment was independently performed at least three times, with at least two technical replicates for each group.

### 2.5 G-protein recruitment assay

Mini-G protein probes translocate to ligand-bound active receptors retaining their specificity (Wan et al., 2018). To measure the ligand-induced recruitment of the Gα_s_, Gα_q_, Gα_i_, and Gα_12/13_, 50 ng DNA of the respective NLuc-tagged mini-G plasmid was co-transfected with 500 ng DNA of GLP-1R GFP or GIPR-GFP per well of a 6-well plate.

### 2.6 cAMP assay

CAMYEL, a cAMP sensor using YFP-Epac-RLuc [23] was utilized to quantify cAMP accumulation with the temporal resolution. Then, 500 ng of CAMYEL DNA was co-transfected with 500 ng of DNA of untagged GLP-1R or GIPR per well in a 6-well plate. The experiments were performed in the absence of 3-isobutyl-1-methylxanthine (IBMX).

### 2.7 GPCR internalization assay

A GPCR internalization assay was established by measuring the loss of baseline resonance energy transfer between an intracellular plasma membrane marker Venus-KRAS and hGLP-1R-RLUC8 or hGIPR-RLUC8 [26]. Then, 500 ng of Venus-KRAS DNA and 300 ng of the respective RLUC8-tagged GPCR DNA were used per well in a 6-well plate.

### 2.8 β-arrestin recruitment assay

Co-localization of β-arrestin1/2-RLUC8 with GLP-1R-GFP or GIPR-GFP was assessed [27]. Fifty ng of β-arrestin1-RLUC8 or β-arrestin2-RLUC8 DNA and 300 ng of GLP-1R-GFP or GIPR-GFP DNA were co-transfected into each well in a 6-well plate.

### 2.9 Endosomal trafficking assay

GPCR endosomal trafficking [28] was assessed by measuring the ligand-stimulated gain in resonance energy transfer between Venus-Rab5/7/11 and hGLP-1R-RLUC8 or hGIPR-RLUC. Then, 100 ng of the respective Venus-Rab subtype DNA and 100 ng of hGLP-1R-RLUC8 or hGIPR-RLUC8 DNA were co-transfected into each well in a 6-well plate.

### 2.10 Endosomal G-protein recruitment assay

Endosomal G-protein recruitment was assessed by bystander BRET via GPCR-induced co-localization of Gα_s_-NLuc with Venus-Rab5/7/11. Then, 300 ng of GLP-1R-untagged or GIPR-untagged DNA, 500 ng of Venus-Rab5/7/11 DNA, and 50 ng of Gα_s_-NLuc DNA were co-transfected per well in a 6-well plate.

### 2.11 HILO microscopy

HEK293T cells were seeded onto 24 mm coverslips (Cat #: 631–1584, VWR, Radnor, PA, USA) and transfected with 500 ng of GLP-1R-GFP or GIPR-GFP over 24 h. HILO image sequences were acquired with a custom-built TIRF microscope (Cairn Research) based on an Eclipse Ti2 (Nikon, Tokyo, Japan) equipped with an EMCCD camera (iXon Ultra, Andor), a 488 nm diode laser, a hardware Perfect Focus System, a TIRF iLas2 module, and a 100× oil-immersion objective (NA 1.49, Nikon). Coverslips were mounted onto metal imaging chambers with a plastic seal and filled with imaging medium (HBSS supplemented with 10 mM of HEPES). The objective and samples were maintained at 37 °C in a heated enclosure. Images were acquired on MetaMorph software (Molecular Devices) using a frame exposure of 50–200 ms with an image acquired before ligand stimulation and a subsequent image taken every 30 s thereafter, up to 20 min. All of the images were analyzed using ImageJ.

### 2.12 Data analysis

Data are represented as means ± S.E.M. Each experiment was independently conducted at least three times, each with at least two technical replicates. E_max_ values were normalized to GLP-1 (7–36 amide) or GIP (1–42). Dose responses were fitted using non-linear regression. pEC50 and EC50 values were calculated using GraphPad Prism 8.0 (GraphPad, San Diego, CA, USA). Statistical analyses were calculated in GraphPad 8.0 using one-way analysis of variance (ANOVA) and corrected with Tukey's or Bonferroni's multiple comparison test. Differences are considered significant with an adjusted p value < 0.05.

## 3 Results

### 3.1 MAR709 and tirzepatide differed from GLP-1R and GIPR mono-agonists in G protein recruitment

**Figure 1:**
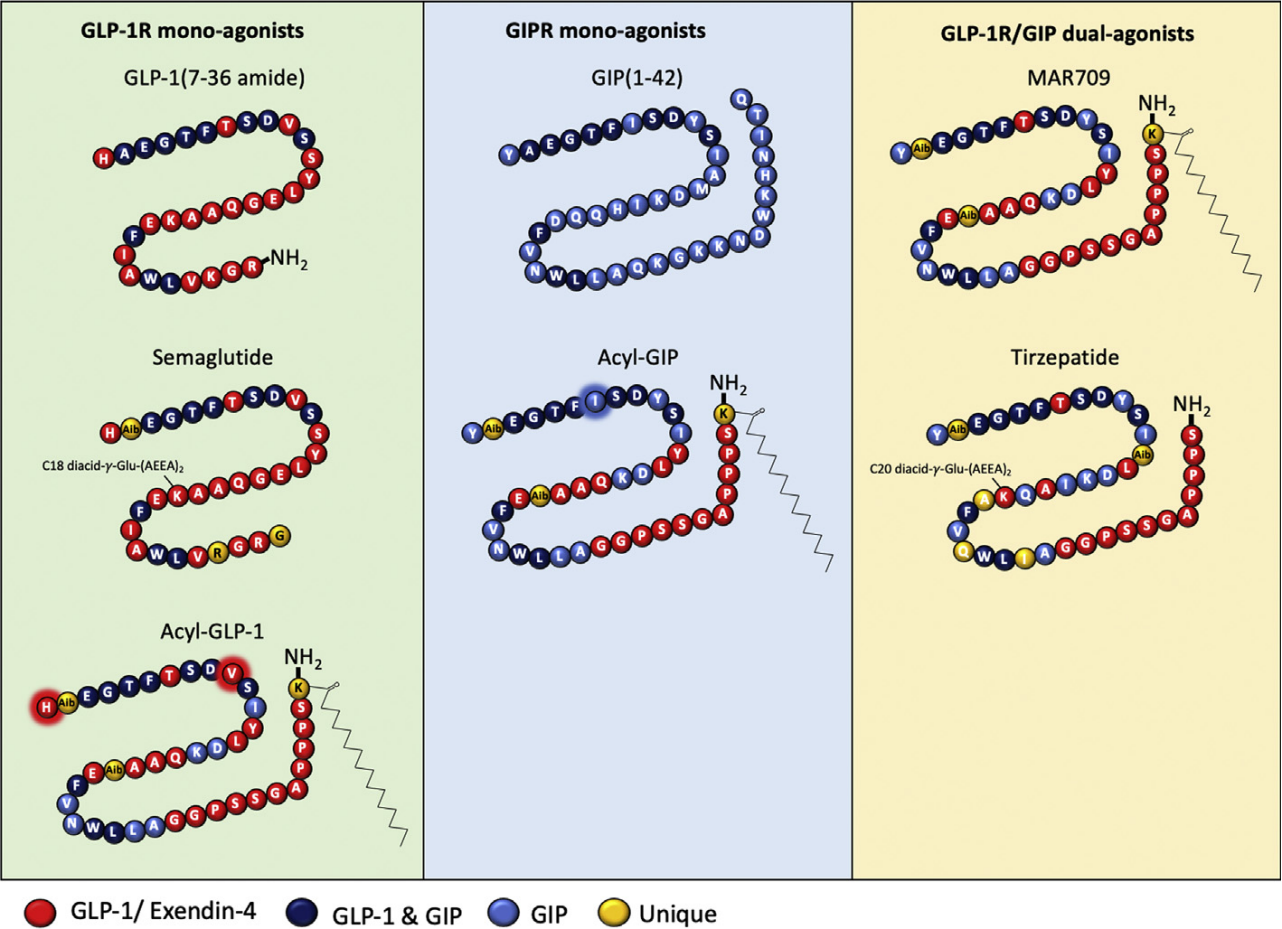
**Schematic and structure of the tested GLP-1R and GIPR ligands**. GLP-1R mono-agonists comprised of human GLP-1 (7–36 amide), semaglutide, and fatty acyl-GLP-1 (a pharmacokinetically-matched His1 and Val10 mutant of MAR709) (left panel). GIPR mono-agonists include human GIP (1–42) and fatty acyl-GIP (a pharmacokinetically matched Ile7 mutant of MAR709) (middle panel). GLP-1/GIP dual-agonist MAR709 and tirzepatide (right panel).

Ligand-induced (1 μM) capacity for receptor G protein recruitment was assessed using bioluminescence resonance energy transfer (BRET)-based technology in HEK293T cells transiently transfected with the respective GFP-tagged receptors and mini-G constructs. The molecules evaluated included the native ligands GLP-1 (7–36 amide) and GIP (1–42), semaglutide (Novo Nordisk, Copenhagen, Denmark), the GLP-1/GIP dual-agonists tirzepatide (Eli Lilly, Indianapolis, IN, USA) and MAR709 (Novo Nordisk, Copenhagen, Denmark), and two molecules (fatty acyl-GLP-1 and fatty acyl-GIP) that are derived from the MAR709 sequence but had been structurally modified via single- or double-point mutations to only activate either GLP-1R or GIPR (Figure 1). A table including the external company identifiers, in-text abbreviations, and amino acid sequence structures of the agonists is available (Supplementary Table 1).

**Figure 2:**
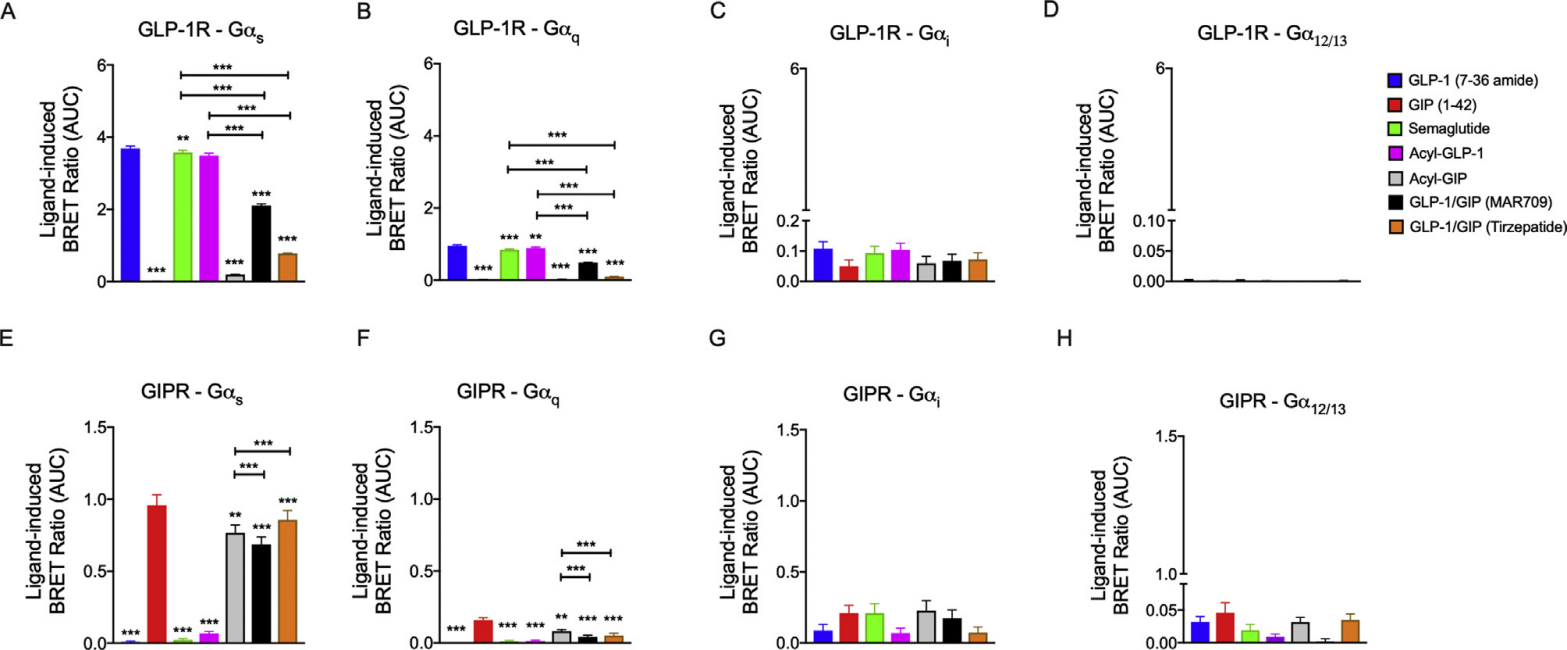
**Ligand-induced G protein recruitment at GLP-1R and GIPR.** Ligand-induced (1 μM) recruitment of Nluc-tagged Gα_s_**(A)**, Gα_q_**(B)**, Gα_i_**(C)**, and Gα_12/13_**(D)** to GFP-tagged GLP-1R in HEK293T cells. Ligand-induced (1 μM) recruitment of Nluc-tagged Gα_s_**(E)**, Gα_q_**(F),** Gα_i_**(G)**, and Gα_12/13_**(H)** with GFP-tagged GIPR^+^ HEK293T cells. The positive iAUC (+iAUC) representation of vehicle and baseline-corrected 30 min response to each agonist is expressed as mean ± SEM. Bonferroni's test, ∗p < 0.05, ∗∗p < 0.005, and ∗∗∗p < 0.0005 using one-way ANOVA vs GLP-1 (7–36 amide) or GIP (1–42). Three independent experiments were performed with at least two technical replicates per group.

In GLP-1R^+^ HEK293T cells, GLP-1 (7–36 amide) strongly recruited Gα_s_ and to a lesser extent Gα_q_, with no meaningful recruitment of Gα_i_ and Gα_12/13_ (Figure 2A–D). GIP (1–42) and fatty acyl-GIP did not stimulate G protein recruitment in GLP-1R^+^ HEK293T cells, while semaglutide and fatty acyl-GLP-1 elicited comparable responses relative to GLP-1 (7–36 amide) (Figure 2A–D). Relative to the GLP-1 mono-agonists, both GLP-1/GIP dual-agonists showed a decreased ability to recruit Gα_s_ and Gα_q_, however, MAR709 demonstrated a higher capacity to recruit Gα_s_ and Gα_q_ compared to tirzepatide (Figure 2A,B). The chimeric structures of MAR709 and tirzepatide did not additionally diversify the G-protein families recruited to the receptor as evidenced by a lack of Gα_i_ and Gα_12/13_ recruitment (Figure 2A–D).

In GIPR^+^ HEK293T cells, native GIP (1–42) predominantly recruited Gα_s_ without meaningful recruitment of Gα_q_, Gα_i_, and Gα_12/13_ (Figure 2E–H). As expected, GLP-1 (7–36 amide), fatty acyl-GLP-1 and semaglutide all showed negligible effects on G protein recruitment in the absence of GLP-1R (Figure 2E–H). Relative to native GIP (1–42), Gα_s_ recruitment following treatment with fatty acyl-GIP and tirzepatide was comparable, but with MAR709 it slightly decreased (Figure 2E).

In summary, the GLP-1 mono-agonists and GLP-1/GIP dual-agonists primarily initiated Gα_s_ recruitment, and to a lesser extent Gα_q_, at the GLP-1R and GIPR. In relation to the GLP-1 mono-agonists, both GLP-1/GIP dual-agonists showed decreased Gα_s_ and Gα_q_ recruitment in GLP-1R^+^ HEK293T cells. In GIPR^+^ cells, tirzepatide led to comparable recruitment of Gα_s_ relative to native GIP while MAR709 showed a slight decrease in Gα_s_ recruitment.

### 3.2 MAR709 and tirzepatide were partial agonists for Gα_s_ recruitment at GLP-1R but full agonists for cAMP production

**Figure 3:**
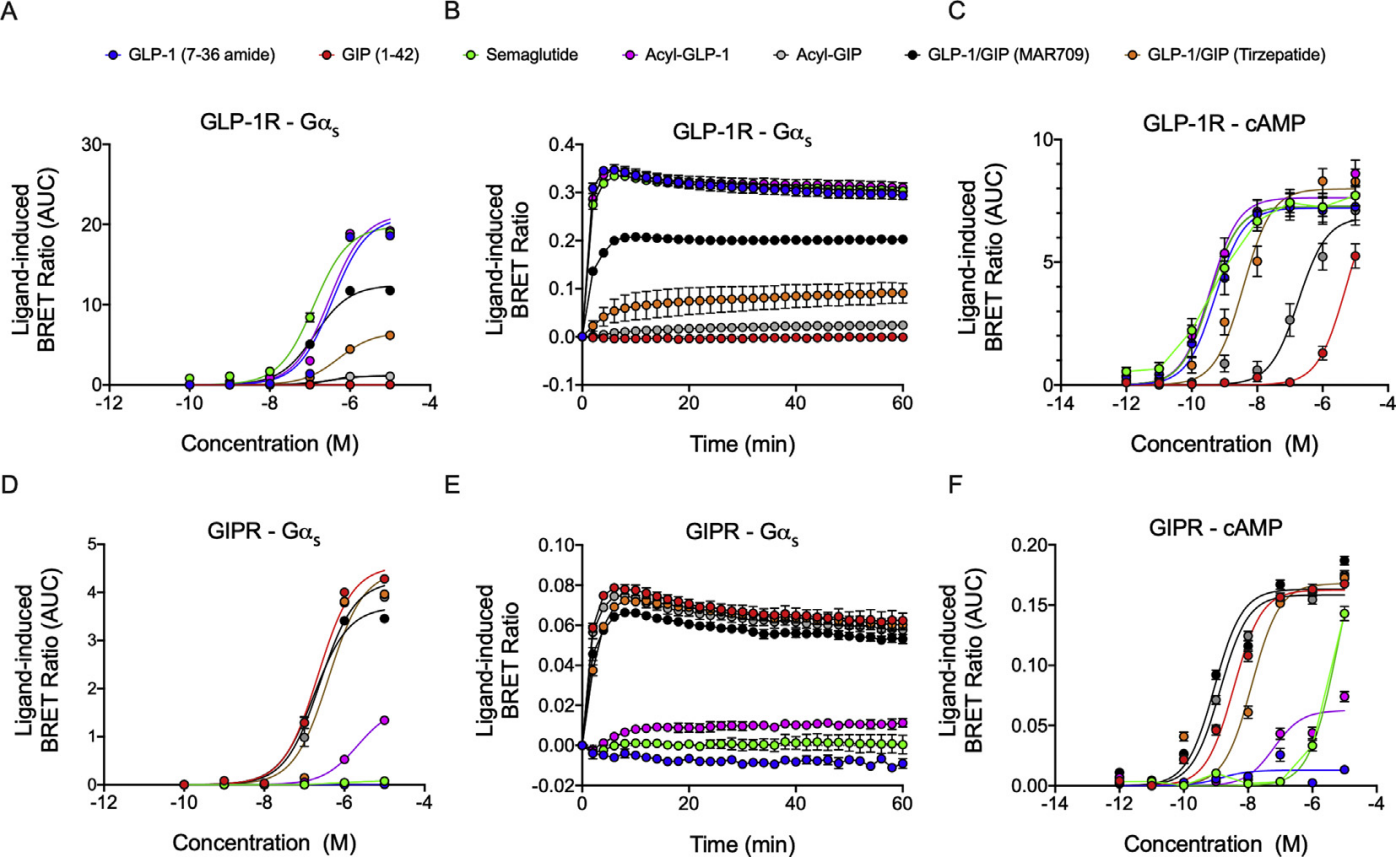
**Dose-dependent effects of ligands on Gα**_**s**_**recruitment and cAMP production.** Dose–response curves **(A)** and temporal resolution (1 μM stimulation) **(B)** of ligand-induced BRET changes between Nluc-tagged Gα_s_ recruitment to GFP-tagged GLP-1R. Dose–response curves of ligand-induced cAMP production GLP-1R^+^ HEK293T cells **(C)**. Dose–response curves **(D)** and temporal resolution (1 μM stimulation) **(E)** of ligand-induced Gα_s_ recruitment to the GIPR. Dose–response curves of ligand-induced cAMP production GIPR^+^ HEK293T cells **(F)**. +iAUC representation of vehicle and baseline-corrected 60 min (Gα_s_ recruitment) or 25 min (cAMP generation) temporal responses to each agonist is expressed as mean ± SEM. Three independent experiments were performed with at least two technical replicates per group.

We next assessed concentration-response dependence in ligand-induced Gα_s_ recruitment and evaluated how this capacity translated to cAMP production. At all of the tested concentrations, semaglutide and fatty acyl-GLP-1 showed comparable Gα_s_ recruitment relative to native GLP-1 (7–36 amide) in GLP-1R^+^ cells (Figure 3A). In line with our previous data (Figure 2A), MAR709 and tirzepatide both acted as partial agonists at the GLP-1R, stimulating a respective 59% and 31% maximal Gα_s_ recruitment (E_max_) relative to GLP-1 (7–36 amide) (Figure 3A and Table 1). This partial agonism was independent of the measurement time after drug exposure (Figure 3B). Interestingly, despite a reduced Gα_s_ recruitment E_max_, the dual-agonists did not differ in cAMP E_max_ compared to the GLP-1 mono-agonists (Figure 3C and Table 1). This was further validated with a CAMYEL sensor saturation assay (Supplementary Figure 4A-C), with ligand responses falling below the saturation limit of the sensor. Hence, despite partial agonism at the level of G protein recruitment to GLP-1R, the dual-agonists remained full agonists when considering cAMP generation. In terms of potency, all of the agonists displayed similar cAMP pEC50 values except for tirzepatide, which was significantly decreased relative to GLP-1 (7–36 amide) (Figure 3C and Table 1).

**Table 1:**
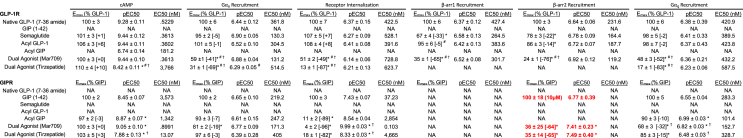
**Maximal (E**_**max**_**) drug effects and affinities at the GLP-1R or GIPR target receptors.** Data were generated in HEK293T cells transiently transfected to express GLP-1R or GIPR. E_max_, pEC50, and EC50 values were generated from dose–response values fitted to sigmoidal curves using a three-parameter non-linear logistic regression. The E_max_ is the maximal response elicited by an agonist and is expressed as % of the maximum response of GLP-1 (7–36 amide) or GIP (1–42). The EC50 is the molar concentration in which an agonist produced half of the maximal response. The pEC50 is the negative logarithm of the EC50. Values are given for Gα_s_ recruitment, cAMP accumulation, receptor internalization, β-arrestin ½, and Gα_q_ recruitment at the GLP-1R and the GIPR. All of the values were derived from the iAUC of a temporal response for each concentration/agonist and are expressed as mean ± SEM from at least 3 independent experiments with at least two technical replicates per group. Statistical significance was determined using one-way ANOVA and corrected with Bonferroni's multiple comparisons test. ∗/^#^/†p < 0.05. ∗ vs GLP-1 (7–36 amide) or GIP (1–42).^#^ vs semaglutide. † vs fatty acyl-GLP-1 or fatty acyl-GIP. NA = no agonism significantly different than zero observed at 1 μM stimulation. Bold red = with significant non-zero agonism at 1 μM stimulation but incomplete curve fit, last value at 10 μM used.

In GIPR^+^ HEK293T cells, we observed a comparable potency and efficacy for Gα_s_ recruitment upon treatment with fatty acyl-GIP and both dual-agonists relative to native GIP (1–42) (Figure 3D and Table 1), which was independent of the measurement time after drug exposure (Figure 3E). MAR709 exhibited a slightly reduced efficacy at the GIPR, stimulating 81% of the Gα_s_ recruitment E_max_ elicited by native GIP (1–42) (Figure 3D and Table 1). For cAMP production, both fatty acyl-GIP and MAR709 displayed a significantly superior pEC50 than that of GIP (1–42), while tirzepatide exhibited a significant 3-fold reduction in potency (Figure 3F and Table 1). Collectively, MAR709 and tirzepatide displayed unique agonism properties at their target receptors, retaining full cAMP efficacy at both the GLP-1R and GIPR despite relatively lower GLP-1R-specific Gα_s_ recruitment efficacy and a slightly reduced relative potency of tirzepatide for cAMP production at the GIP receptor.

### 3.3 MAR709 and tirzepatide showed decreased receptor internalization relative to GLP-1R and GIPR mono-agonists

**Figure 4:**
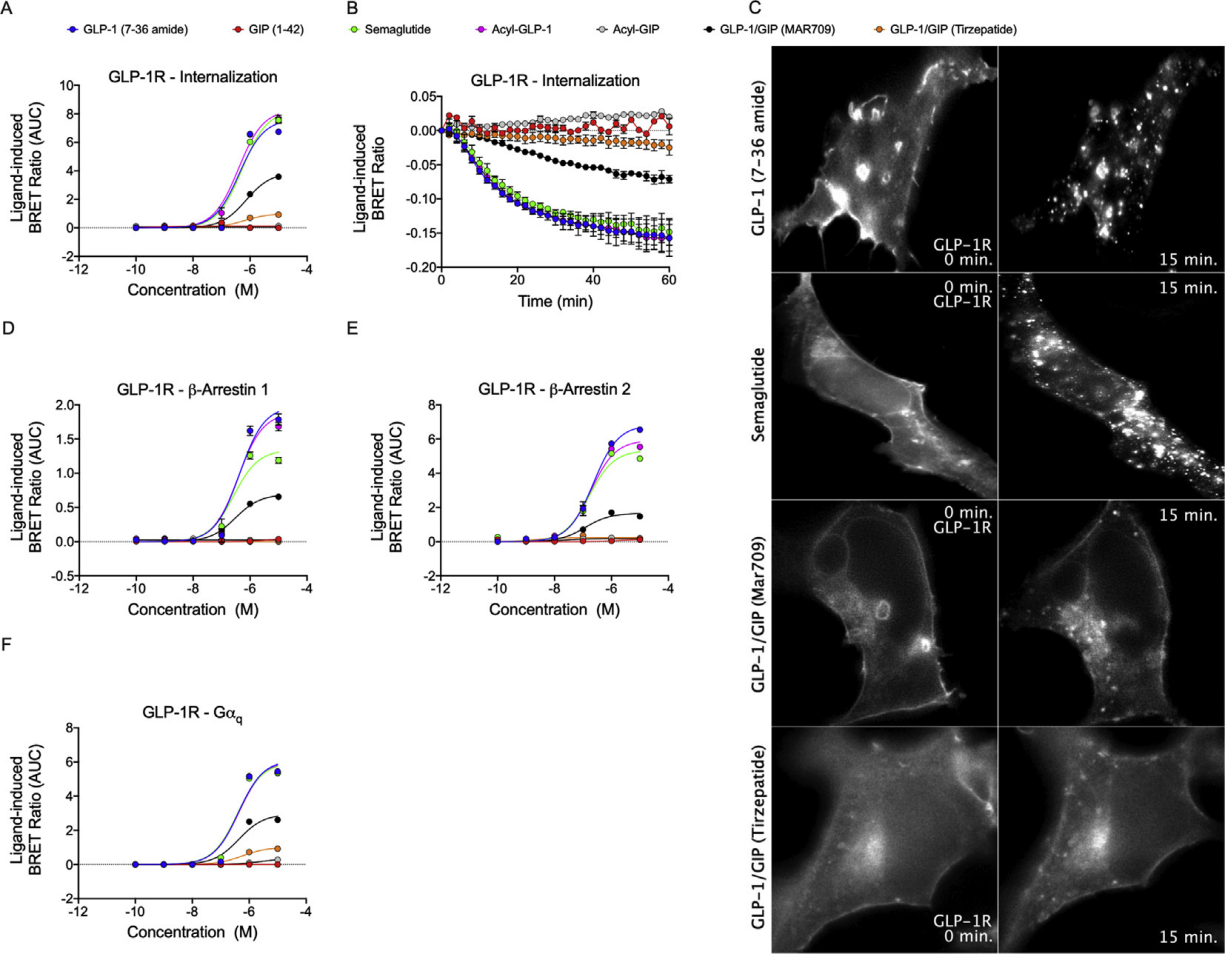
**Ligand-induced GLP-1R internalization.** Dose–response curves **(A)** and temporal resolution (1 μM stimulation) **(B)** of ligand-induced hGLP-1-Rluc8 internalization as measured by loss of BRET with plasma membrane marker Venus-KRAS. Live HILO imaging of GLP-1R-GFP internalization in HEK293T cells at baseline and approximately 15 min after drug (1 μM) treatment (representative image from n = 4 experiments) **(C)**. Dose–response curves for β-arrestin 1-Rluc8 **(D)**, β-arrestin 2-Rluc8 **(E)**, and Gα_q_-Nluc recruitment **(F)** to GLP-1R-GFP. The + iAUC representation of vehicle and baseline-corrected 60 min (GLP-1R internalization and Gα_q_ recruitment) or 30 min (β-arrestin1/2 recruitment) temporal response to each agonist is expressed as mean ± SEM. Three independent experiments were performed with at least two technical replicates per group.

We next assessed ligand-induced receptor internalization and the recruitment of β-arrestin and Gα_q_. In hGLP-1R-Rluc8^+^ HEK293T cells, semaglutide and fatty acyl-GLP-1 showed similar receptor internalization dynamics relative to GLP-1 (7–36 amide) (Figure 4A,B). However, both MAR709 and tirzepatide showed strikingly decreased receptor internalization compared to the tested GLP-1R mono-agonists (Figure 4A,B). Relative to GLP-1 (7–36 amide), the maximal ligand-induced GLP-1R internalization (E_max_) of MAR709 and tirzepatide was 51% and 13%, respectively (Figure 4A,B and Table 1). Likewise, decreased internalization of GLP-1R was also observed upon treatment of hGLP-1R-Rluc8^+^ Min6 cells with MAR709 and tirzepatide relative to GLP-1 (7–36 amide) and GLP-1 mono-agonists (Supplementary Figure 5A-C). No significant differences were observed in the pEC50 values of the tested ligands in HEK293T cells. Decreased receptor internalization mediated by MAR709 and tirzepatide was also confirmed using live cell HILO microscopy in HEK293T cells expressing GLP-1R-GFP (Figure 4C). While treatment with GLP-1 (7–36 amide) and semaglutide initiated rapid internalization of GLP-1R-GFP, MAR709 and tirzepatide showed the persistent presence of the ligand–receptor complex at the plasma membrane with strikingly less trafficking into the cytosol (Figure 4C). These data collectively demonstrated that MAR709 and tirzepatide differed from the GLP-1R mono-agonists in that they showed prolonged receptor presence at the cell surface and reduced receptor internalization.

GLP-1R recruitment of β-arrestin 1/2 (β-arr1/2) has been shown to influence receptor trafficking and enhance extracellular signal-regulated kinase 1/2 (ERK1/2) signaling [29]. In GLP-1R^+^ HEK293T cells, semaglutide stimulated 67% and 78% of the β-arr1 and β-arr2 recruitment E_max_ elicited by GLP-1 (7–36 amide), while fatty acyl-GLP-1 elicited a slightly reduced 86% of β-arr2 (Figure 3D,E and Table 1). A pronounced reduction in β-arrestin recruitment efficacy with the dual-agonists was observed, in which treatment with MAR709 led to 35% and 24% of the GLP-1 (7–36 amide) β-arr1 and β-arr2 recruitment E_max_ (Figure 4D,E and Table 1), while no measurable response for either β-arr1 or β-arr2 was seen with tirzepatide (Figure 4D,E and Table 1).

GLP-1R recruitment of Gα_q_ has been proposed to regulate GLP-1R internalization via an ERK1/2 pathway [30]. In line with this data and our demonstration of decreased GLP-1R internalization upon treatment with MAR709 and tirzepatide (Figure 4A–C), we saw a less efficacious Gα_q_ recruitment response to the GLP-1R upon treatment with MAR709 and tirzepatide, in which 48% and 17% of the GLP-1 (7–36 amide) E_max_ was achieved, respectively (Figure 4F and Table 1).

**Figure 5:**
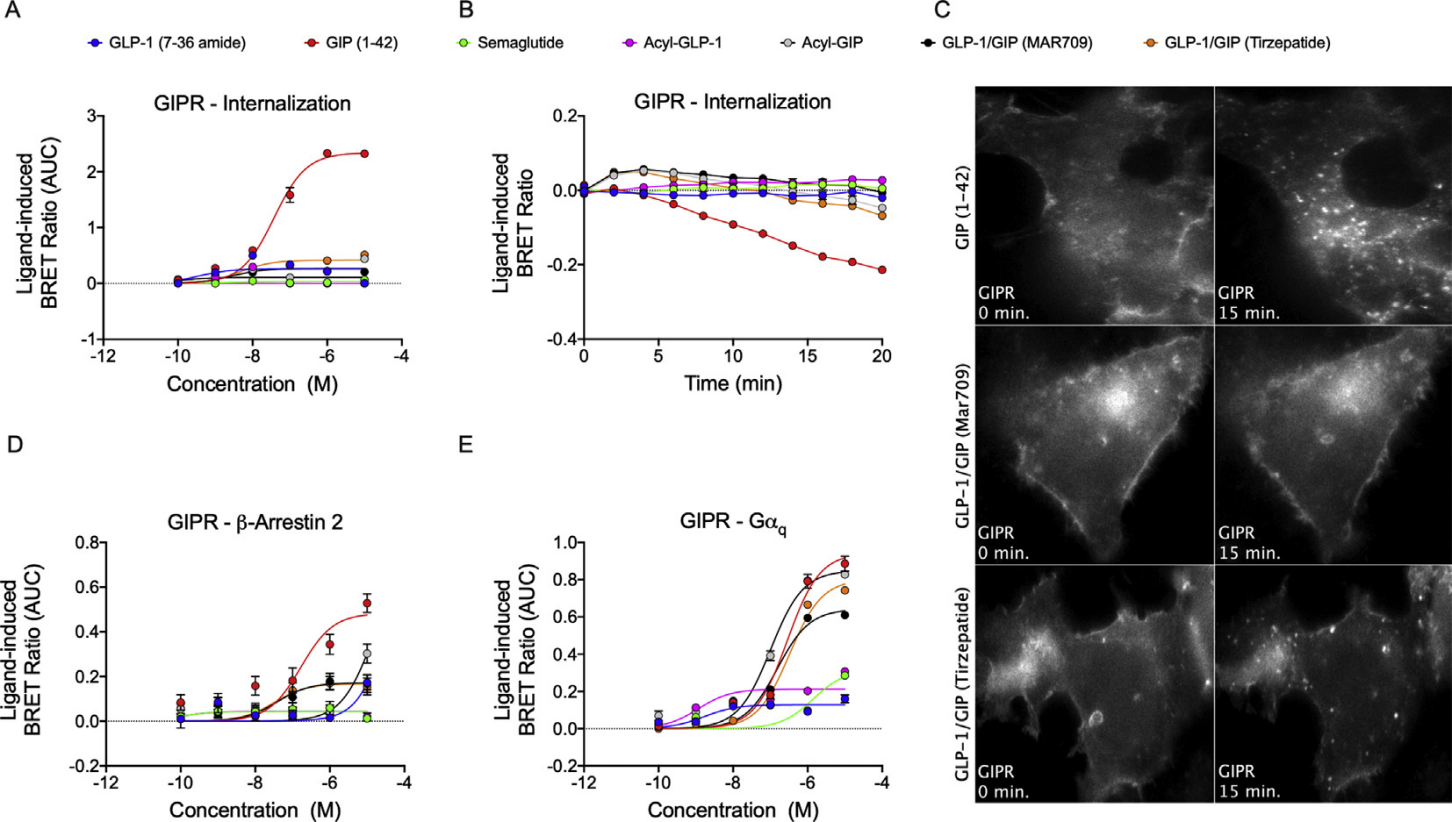
**Ligand-induced GIPR internalization.** Dose–response curves **(A)** and temporal resolution (1 μM stimulation) **(B)** of ligand-induced hGIPR-Rluc8 internalization. Live HILO imaging of GIPR-GFP internalization in HEK293T cells at baseline and approximately 15 min after drug (1 μM) treatment **(C)**. Dose–response curves for β-arrestin 2-Rluc8 **(D)** and Gα_q_-Nluc recruitment **(E)**. The + iAUC representation of vehicle and baseline-corrected 20 min (GIPR internalization), 30 min (β-arrestin1/2 recruitment), or 60 min (Gα_q_ recruitment) temporal response to each agonist is expressed as mean ± SEM. Three independent experiments were performed with at least two technical replicates per group.

In hGIPR^+^ HEK293T cells, we observed sustained receptor internalization induced by GIP (1–42) but no meaningful internalization following treatment with either fatty acyl-GIP, the GLP-1 mono-agonists, or the dual-agonists (Figure 5A,B). In detail, MAR709 and tirzepatide stimulated 4% and 18% of the GIP (1–42) receptor internalization E_max_ (Figure 5A,B and Table 1). Reduced capacity of the dual-agonists for GIPR internalization was also confirmed visually through live-cell microscopy. Fifteen minutes after compound administration, GIP (1–42) showed a high dissolution of the GIPR-GFP-defined plasma membrane border with greater punctate structure formation in the cytosol, while neither MAR709 nor tirzepatide evoked a similar dynamic (Figure 5C).

Unlike β-arr2, β-arr1 has been shown to lack a functional role in GIPR internalization and trafficking [31]. Relative to GIP (1–42) at the maximal concentration of 10 μM, a 36% and 35% β-arr2 recruitment response was observed in cells treated with MAR709 or tirzepatide (Figure 5D and Table 1). A true comparison between GIPR-β-arr2 agonist E_max_ was not possible due to an incomplete curve fit for GIP (1–42). However, these data collectively suggested that reduced β-arrestin 2 recruitment by the dual-agonists may have had a functional correlation in the observed reduction in GIPR internalization or trafficking by these molecules.

Relative to the Gα_q_ recruitment E_max_ for GIP (1–42), treatment with fatty acyl-GIP displayed a similar efficacy while MAR709 and tirzepatide exhibited 68% and 85% of the maximal response (Figure 5E and Table 1).

In summary, these data showed that MAR709 and tirzepatide both differed from the native peptides, semaglutide, and the PK-matched receptor mono-agonists (fatty acyl-GIP and fatty acyl-GLP-1) in that they showed reduced internalization and decreased β-arrestin and Gα_q_ recruitment at both target receptors.

### 3.4 MAR709 and tirzepatide induced differential endosomal receptor trafficking relative to GLP-1R and GIPR mono-agonists

**Figure 6:**
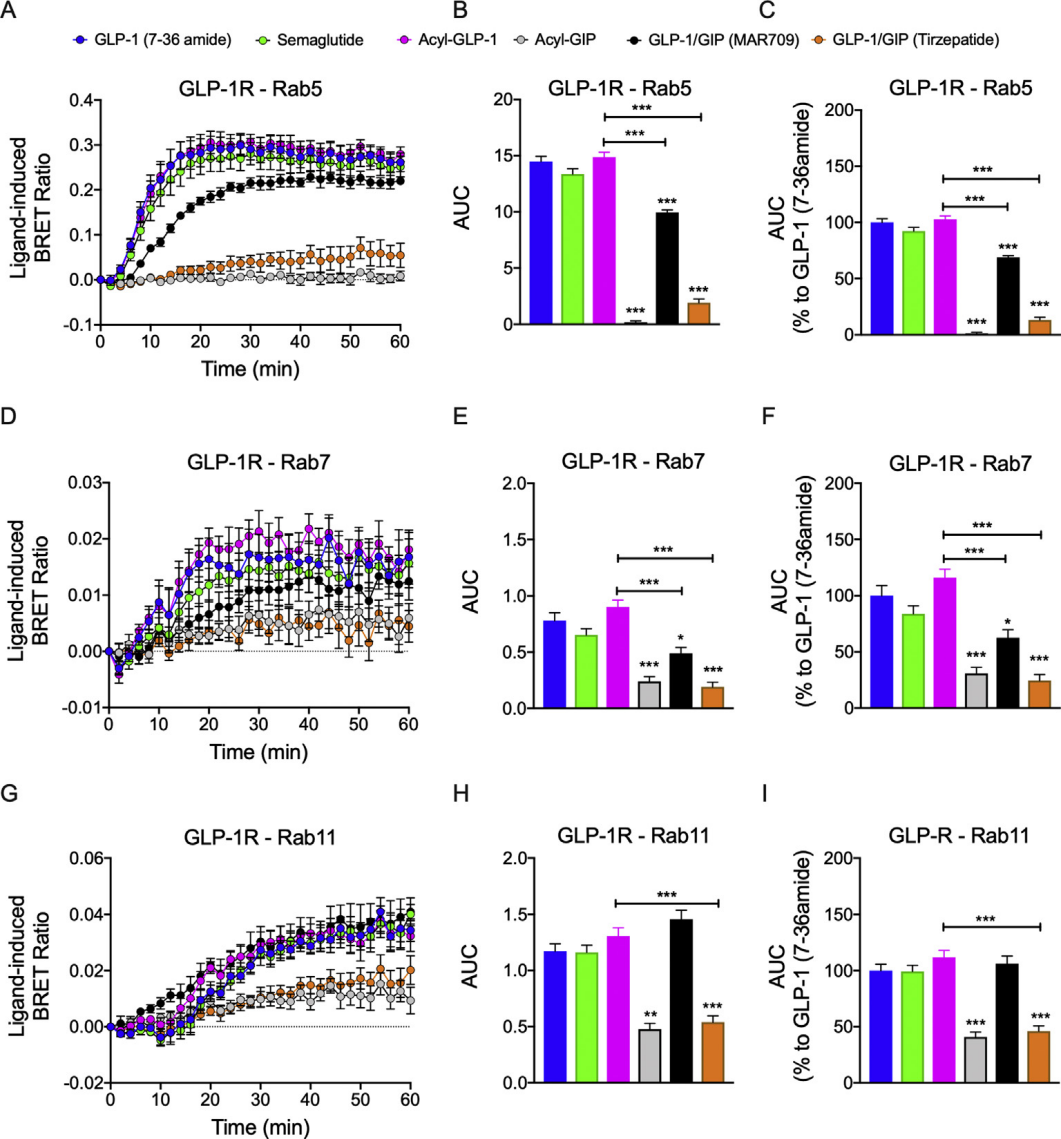
**Ligand-induced GLP-1R endosomal trafficking.** Ligand-induced co-localization of GLP-1R-Rluc8 with Venus-Rab5 early endosomes **(A**–**C)**, Venus-Rab7 late endosomes **(D**–**F)**, and Venus-Rab11 recycling endosomes **(G**–**I)**. The + iAUC representation of vehicle and baseline-corrected temporal response to each agonist is expressed as mean ± SEM. Bonferroni's test, ∗p < 0.05, ∗∗p < 0.005, and ∗∗∗p < 0.0005 using one-way ANOVA vs GLP-1 (7–36 amide). Six independent experiments were performed with at least two technical replicates per group.

**Supplementary Figure 1:**
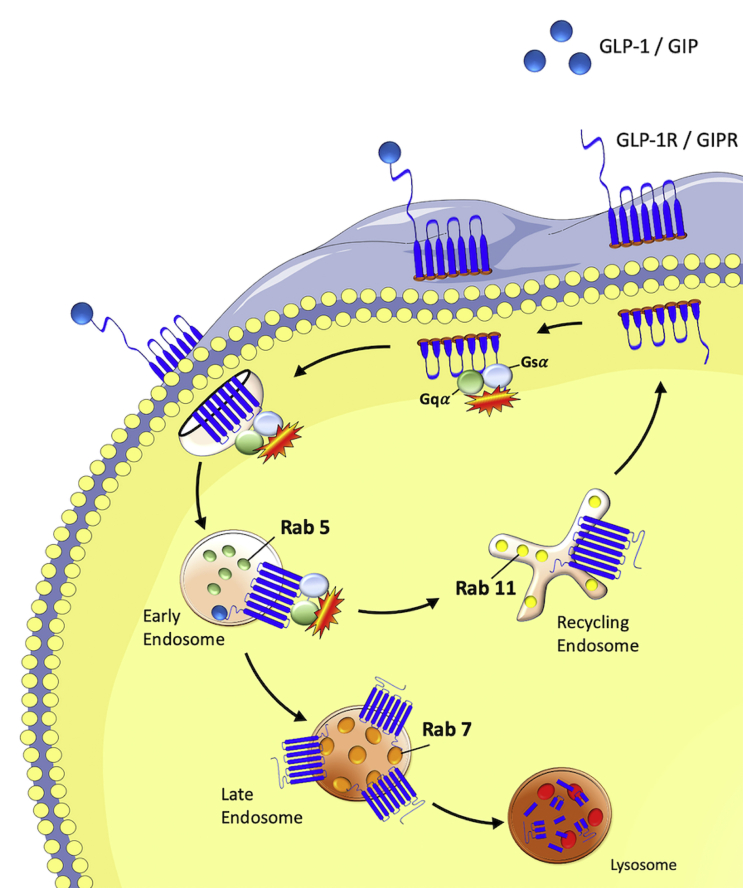
**Schematic of the endosomal marker associated with GLP-1R/GIPR internalization and trafficking.** Markers indicative of endosomal trafficking include Rab5 (early endosome), Rab7 (late endosome), and Rab11 (recycling endosome).

**Supplementary Figure 2:**
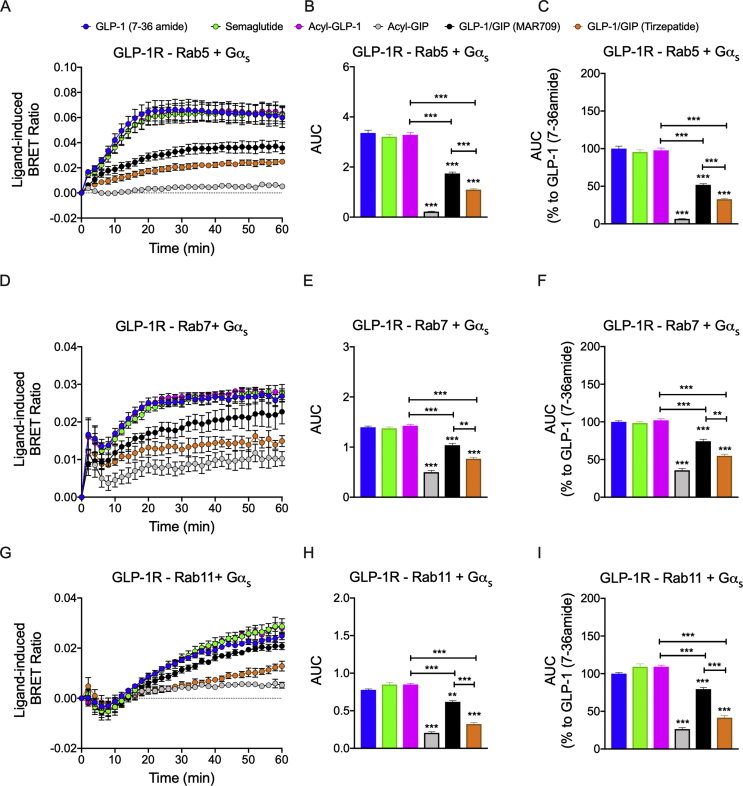
**Co-localization of GLP-1R with Gα**_**s**_**and Rab5, Rab7, or Rab11 in GLP-1R**^**+**^**HEK293T cells.** Ligand-induced HEK293T GLP-1R-mediated proximal co-localization of Nluc-tagged Gα_s_ with Venus-tagged Rab5 **(A-C)**, Gα_s_ and Rab7 **(D-F)**, or Gα_s_ and Rab11 **(G-I)** over 60 min. The +iAUC representation of vehicle and baseline-corrected temporal response to each agonist expressed as mean ± SEM. Bonferroni’s test, ∗p < 0.05, ∗∗p < 0.005, and ∗∗∗p < 0.0005 using one-way ANOVA vs GLP-1 (7-36 amide). Three independent experiments were performed with at least two technical replicates per group.

We next evaluated endosomal trafficking of the ligand-receptor complexes by assessing the co-localization of hGLP-1R-Rluc8 with Venus-tagged markers indicative of early endosomes (Rab5), late endosomes (Rab7), or recycling endosomes (Rab11) (Supplementary Figure 1). Consistent with our previous results showing decreased internalization of GLP-1R and GIPR by the dual-agonists (Figure 4A–C and Figure 5A–C), 1 μM stimulation with MAR709 or tirzepatide resulted in 68% and 13% of the total GLP-1R Rab5 co-localization elicited by GLP-1 (7–36 amide) (Figure 6A–C). Similar patterns were also observed when assessing total Gα_s_ recruitment to GLP-1R^+^ Rab5^+^ endosomes (Supplementary Figure 2A-C). No difference in Rab5 co-localization was observed between GLP-1 (7–36 amide), semaglutide, and fatty acyl-GLP-1 (Figure 6A–C). In a hGLP-1R-Rluc8^+^ min6 β cell model, tirzepatide likewise stimulated reduced co-localization of GLP-1R into Rab5^+^ endosomes compared to GLP-1 (7–36 amide) and GLP-1 mono-agonists (Supplementary Figure 5D-F). Within HEK293T cells, co-localization of GLP-1R with Rab7 positive (late) endosomes was reduced, with MAR709 and tirzepatide stimulating 62% and 24% of the response of GLP-1 (7–36 amide) (Figure 6D–F). This pattern was replicated in ligand-induced Gα_s_ recruitment to GLP-1R^+^ Rab7^+^ endosomes (Supplementary Figure 2D-F). Notably, differences in GLP-1R co-localization with Rab11-positive recycling endosomes were insignificant between treatments of MAR709 and GLP-1 (7–36 amide), but treatment with tirzepatide decreased by 54% (Figure 6G–I). Despite substantial Gα_s_ recruitment to Rab11^+^ endosomes, endosomal Gα_s_ recruitment by MAR709 was significantly reduced compared to GLP-1 (7–36 amide) (Supplementary Figure 2F-I). Regarding the Min6 cell model, due to either a lack of BRET signals or the requirement for improved detection sensitivity, replication of ligand-induced GLP-1R co-localization with Rab7-and Rab11-positive endosomes was not observable for any agonist (Supplementary Figure 5G-J). In HEK293T cells, the general agonist relationship between the AUC of GLP-1R endosomal co-localization and endosomal G-protein recruitment was positively linear, in which greater endosomal trafficking was associated with greater Gα_s_ recruitment to the endosomal sub-compartment (Supplementary Figure 6A-C). In summary, these data indicated that MAR709 not only induced less GLP-1R co-localization into early and late endosomes but also comparably incorporated GLP-1R into Rab11^+^ recycling endosomes to that of GLP-1 (7–36 amide) and semaglutide in HEK293T cells.

**Figure 7:**
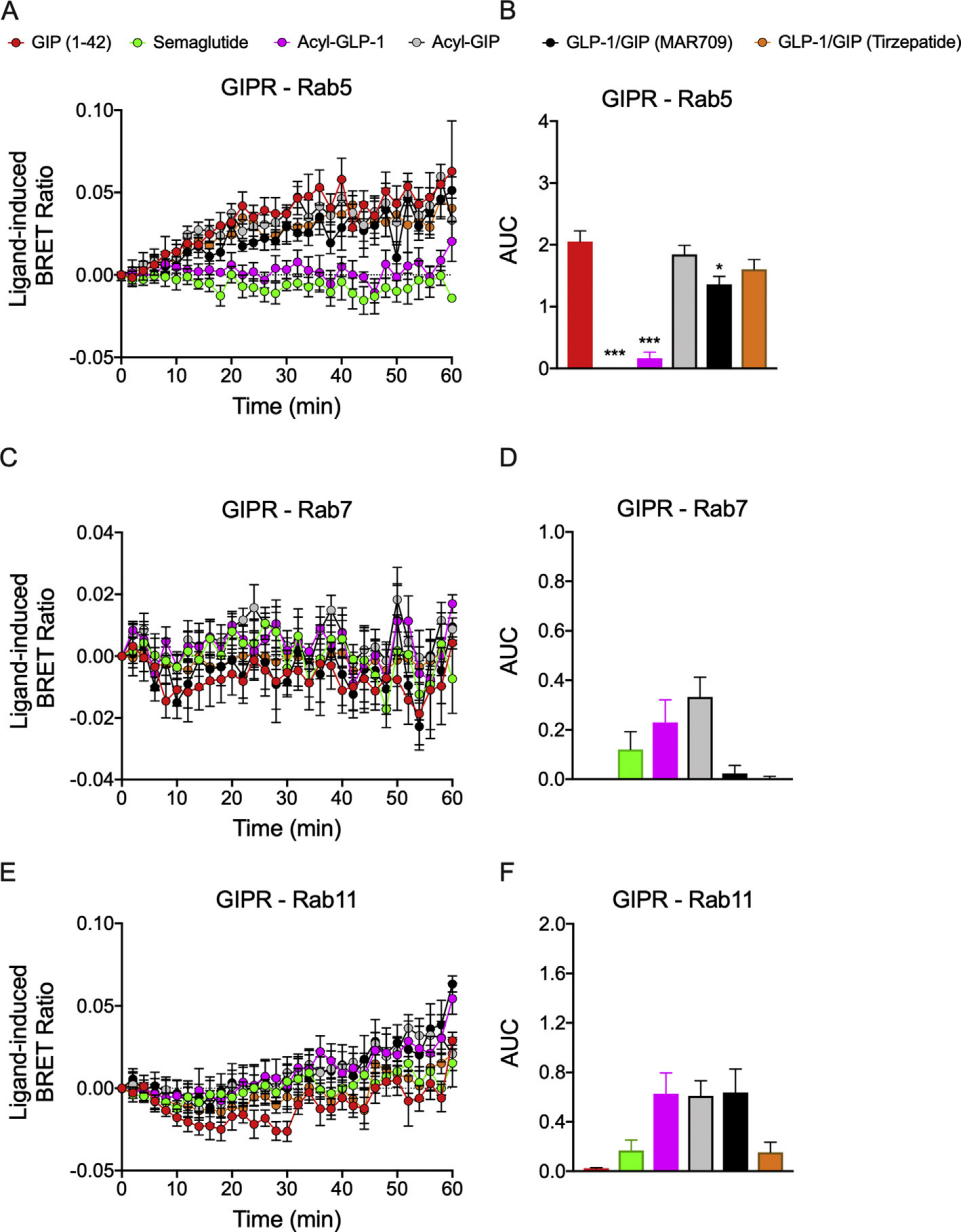
**Ligand-induced GIPR endosomal trafficking.** Ligand-induced co-localization of GIPR with Venus-Rab5^+^ early endosomes **(A** and **B)**, Venus-Rab7^+^ late endosomes **(C** and **D)**, and Venus-Rab11^+^ recycling endosomes **(E** and **F)**. The + iAUC representation of vehicle and baseline-corrected temporal response to each agonist is expressed as mean ± SEM. Bonferroni's test, ∗p < 0.05, ∗∗p < 0.005, and ∗∗∗p < 0.0005 using one-way ANOVA vs GIP (1–42). Six independent experiments were performed with at least two technical replicates per group.

**Supplementary Figure 3:**
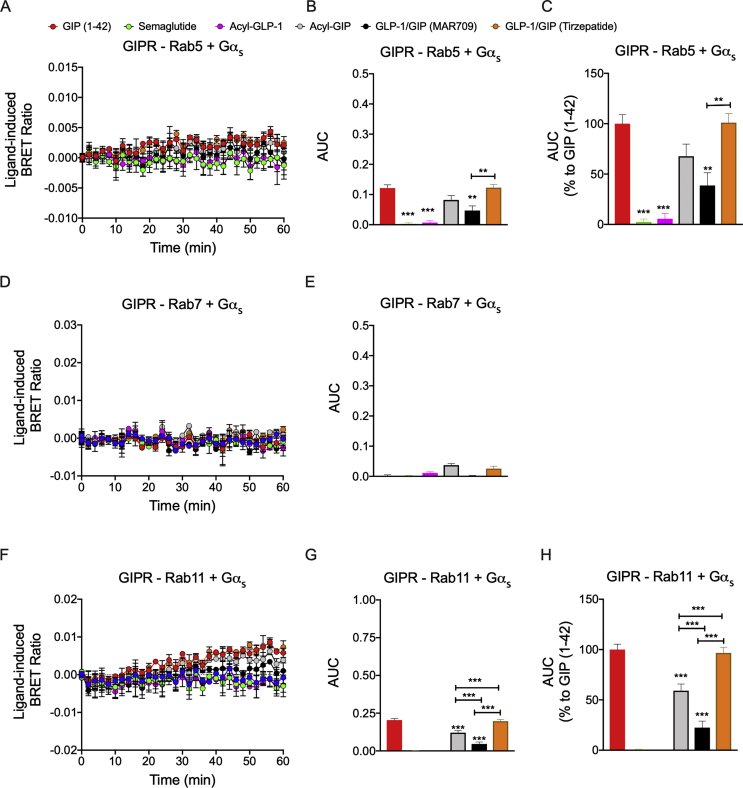
**Co-localization of GIPR with Gα**_**s**_**and Rab5, Rab7, or Rab11 in GIPR**^**+**^**HEK293T cells.** Ligand-induced HEK293T GIPR-mediated proximal co-localization of Nluc-tagged Gα_s_ and Venus-Rab5 **(A-C)**, Gα_s_ and Rab7 **(D and E)**, or Gα_s_ and Rab11 **(F-H)** over 60 min. The +iAUC representation of vehicle and baseline-corrected temporal response to each agonist is expressed as mean ± SEM. Bonferroni’s test, ∗p < 0.05, ∗∗p < 0.005, and ∗∗∗p < 0.0005 using one-way ANOVA vs GIP (1-42). Three independent experiments were performed with at least two technical replicates per group.

**Supplementary Figure 4:**
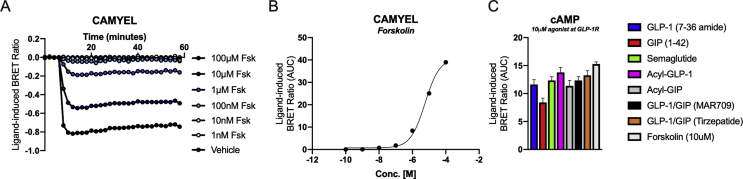
**CAMYEL cAMP sensor evidenced lack of saturation at 10 μM of GLP-1R agonist.** Time course **(A)** and fitted dose-response curve **(B)** of cAMP-positive control forskolin as measured by the intracellular sensor CAMYEL in HEK293T cells. Stimulation with 10 μM of agonist or forskolin in GLP-1R+ HEK293T cells **(C)**. The +iAUC representation of vehicle and baseline-corrected temporal response to each agonist is expressed as mean ± SE. Three independent experiments were performed with at least two technical replicates per group.

**Supplementary Figure 5:**
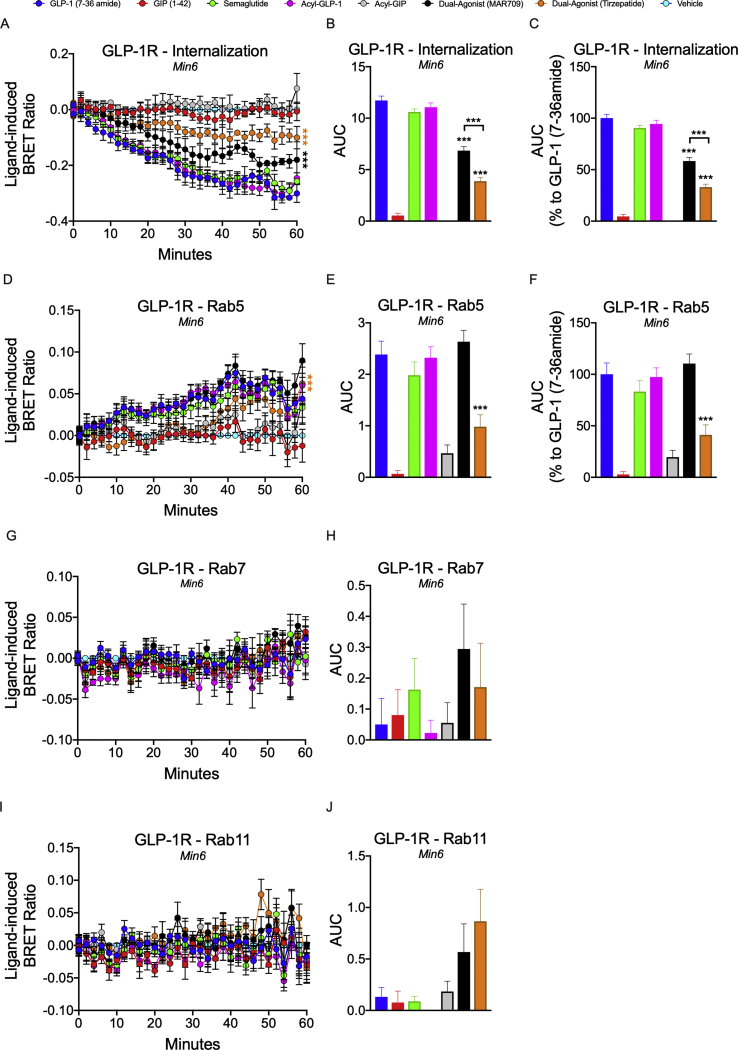
**GLP-1R internalization and Rab5 co-localization was differentially effected by the dual-agonists (1μM) in Min6 cells.** Agonist-induced internalization of hGLP-1R-Rluc8 in Min6 cells for 60 min (A). Agonist-induced hGLP-1R-Rluc8 co-localization with Venus-Rab5^+^ (B), Venus-Rab7^+^ (C), and Venus-Rab11^+^ (D) endosomes in Min6 cells for 60 min. Temporal response for each agonist is expressed as mean ± SE. Tukey's test, ∗p < 0.05, ∗∗p < 0.005, and ∗∗∗p < 0.0005 using one-way ANOVA vs GLP-1 (7-36 amide). Six independent experiments were performed with at least two technical replicates per group.

**Supplementary Figure 6:**
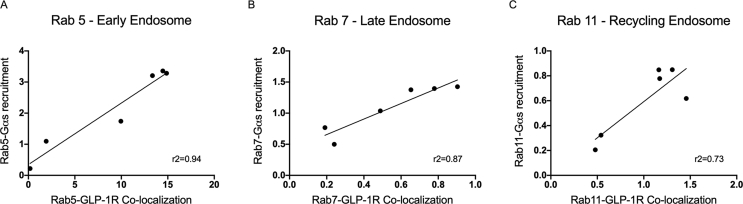
**Degree of Rab-specific GLP-1R co-localization was positively associated with the degree of GLP-1R-mediated endosomal Gαs recruitment in HEK293T cells.** Linear regression between Gα_s_ recruitment and GLP-1R co-localization at the Rab5- (A), Rab7- (B), and Rab11-positive (C) endosomal compartments.

In GIPR^+^ HEK293T cells, GIPR co-localization into Rab5^+^ endosomes was similar upon treatment with GIP (1–42), fatty acyl-GIP, and tirzepatide; however, MAR709 achieved approximately 66% of this response (Figure 7A,B). This pattern was also seen in Gα_s_ recruitment to Rab5^+^ endosomes. No meaningful co-localization was detected with GIPR at either Rab7 or Rab11 (Figure 7C–F). The lack of receptor co-localization with Rab7^+^ and Rab11^+^ endosomes was similarly associated with a lack of endosomal Gα_s_ recruitment (Supplementary Figure 3D-H). Discrepancies between GIPR Rab5^+^ co-localization and the lack of GIP receptor internalization by the dual-agonists likely reflected methodological differences and/or lack of Rab5^+^ early endosome scission from the plasma membrane.

## 4 Discussion

Our data showed that the GLP-1/GIP dual-agonists MAR709 and tirzepatide differed from the GLP-1R and GIPR mono-agonists in terms of G protein recruitment, target receptor internalization, and endosomal trafficking. Although both dual-agonists showed delayed internalization at both target receptors, MAR709 but not tirzepatide induced comparable GLP-1R accumulation into Rab11^+^ recycling endosomes to that of GLP-1 (7–36 amide) and semaglutide.

Both MAR709 and tirzepatide exhibited reduced Gα_s_ recruitment to the GLP-1R relative to GLP-1 (7–36 amide) while retaining full-agonist capacity for cAMP, likely an advantageous effect of signal amplification systems. Similarly, both MAR709 and tirzepatide evidenced full agonism for cAMP at the GIPR, but only MAR709 displayed characteristics of partial agonism with a slight reduction in Gα_s_ recruitment efficacy. These data together were in line with previously established reports [19]. Since MAR709 and tirzepatide showed 100% sequence homology at positions 1–12, the observed differences between MAR709 and tirzepatide apparently resulted from sequence substitutions at positions 13–27 of the peptides or from the size and location of fatty acylation. The aforementioned differences in total and endosomal Gα_s_ recruitment may play a role in the endosomal sorting of the internalized receptor to Rab7^+^/lysosomal pathways [32].

GLP-1R internalization is primarily caveolin-1/dynamin dependent [33], mediated by Gα_q_ signaling [30], and does not require but is influenced by β-arrestin [16,34]. GLP-1R internalization has been linked to the degree of cellular desensitization and insulin secretion in vitro [16,35]. Tirzepatide has previously been shown to elicit reduced GLP-1R internalization relative to native GLP-1 [36]. Whether this effect also holds true for other dual-agonists has yet to be demonstrated. Both dual-agonists evaluated herein retained a higher presence of GLP-1R at the plasma membrane relative to the tested GLP-1R mono-agonists and similarly displayed corresponding partial agonism for β-arrestin 1, β-arrestin 2, and Gα_q_ recruitment to the GLP-1R. A Phe1 substitution within an exendin-4 sequence has previously been described to reduce GLP-1R internalization and β-arrestin recruitment [16]. In line with this, reduced internalization is also observed with a (phenolic) Tyr1 present in the MAR709 and tirzepatide amino acid sequences.

Both dual-agonists showed minimal GIPR internalization relative to GIP (1–42). Yet, both GIP (1–42) and tirzepatide elicited equal GIPR incorporation into Rab5+ early endosomes. Reasons for the discrepancy might have originated in the methodology of how internalization was assessed.

Ligand-induced GLP-1R endosomal trafficking has not yet been fully elucidated. We showed that MAR709 did not differ from GLP-1 (7–36 amide) and semaglutide in terms of eliciting GLP-1R co-localization with Rab11+ recycling endosomes. Whether this was a consequence primarily of internalized receptor diverting into recycling pathways or whether increased Rab11 co-localization induced by MAR709 was supplemented with recruitment of GLP-1R from the biosynthetic pathway has yet to be established. Given the low rate of GLP-1R internalization and incorporation into Rab5+ and Rab7+ endosomes, MAR709's high capacity for Rab11+ co-localization and its biased signaling profile demonstrated unique spatiotemporal pharmacology at the GLP-1R that may facilitate potential attributes of cellular sensitization. A caveat to the receptor trafficking experiments was the limited potential for aberrant Venus-Rab localization into non-specific endosomal compartments occurring from over-expression associated changes in Rab trafficking patterns. Additionally, transferability of these findings to physiologically relevant β cells was restricted to the min6 β cell model, and hence represents a limitation of this work.

Despite favoring GIPR over the GLP-1R, tirzepatide showed comparable efficacy and potency relative to MAR709 at multiple signaling pathways connected to the GIPR, with the exception of cAMP pEC50 in which MAR709 exhibited higher potency. At the GLP-1R, MAR709 displayed higher Gα_s_/Gα_q_ signaling, receptor internalization, and β-arrestin recruitment relative to tirzepatide despite still acting as a partial agonist in each of these categories. In addition, MAR709 elicited a disproportional incorporation of the GLP-1R into Rab11^+^ recycling endosomes. Collectively, our data showed that MAR709 and tirzepatide differed from the tested receptor agonists in G protein recruitment, receptor internalization, and endosomal trafficking, which together supports the hypothesis that biased agonism of these molecules might contribute to their beneficial metabolic action profile.

## Author contributions

A.N. and S.O’B. co-conceptualized the project, designed and performed the experiments, analyzed and interpreted the data, and wrote the manuscript. M.B., G.G., and M.K. conducted the experiments and analyzed and interpreted the data. P.K. performed peptide synthesis. A.Z., M.K., K.S., R.D.D., M.H.T., and B.F. co-conceptualized the project, interpreted the data, and revised the manuscript critically. D.C. and T.D.M. conceptualized and supervised the experiments, analyzed and interpreted the data, and wrote the manuscript.

## Figures and Tables

**Supplementary Table 1:**
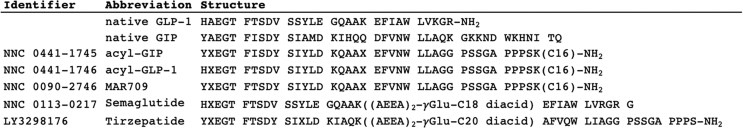
**Structure and identifiers of GLP-1 and GIP mono- and dual-agonists.** Amino acid sequence, in-paper abbreviations, and external identifiers of the GLP-1 and GIP-derived compounds used.
